# Clinical, Laboratory, and Management Profile in Patients of Liver Abscess from Northern India

**DOI:** 10.1155/2014/142382

**Published:** 2014-06-04

**Authors:** Soumik Ghosh, Sourabh Sharma, A. K. Gadpayle, H. K. Gupta, R. K. Mahajan, R. Sahoo, Naveen Kumar

**Affiliations:** ^1^Department of Medicine, PGIMER, Dr. RML Hospital, New Delhi 110001, India; ^2^Department of Microbiology, PGIMER, Dr. RML Hospital, New Delhi 110001, India

## Abstract

*Objective*. To describe the clinical profile, microbiological aetiologies, and management outcomes in patients with liver abscess. *Methods*. A cross-sectional study was conducted from May, 2011, to April, 2013, on 200 consecutive liver abscess patients at PGIMER and Dr. RML Hospital, New Delhi. History, examination, and laboratory investigations were recorded. Ultrasound guided aspiration was done and samples were investigated. Chi-square test and multivariate regression analysis were performed to test association. *Results*. The mean age of patients was 41.13 years. Majority of them were from lower socioeconomic class (67.5%) and alcoholic (72%). The abscesses were predominantly in right lobe (71%) and solitary (65%). Etiology of abscess was 69% amoebic, 18% pyogenic, 7.5% tubercular, 4% mixed, and 1.5% fungal. Percutaneous needle aspiration was done in 79%, pigtail drainage in 17%, and surgical intervention for rupture in 4% patients. Mortality was 2.5%, all reported in surgical group. Solitary abscesses were amoebic and tubercular whereas multiple abscesses were pyogenic (*P* = 0.001). Right lobe was predominantly involved in amoebic and pyogenic abscesses while in tubercular abscesses left lobe involvement was predominant (*P* = 0.001). *Conclusions*. The commonest presentation was young male, alcoholic of low socioeconomic class having right lobe solitary amoebic liver abscess. Appropriate use of minimally invasive drainage techniques reduces mortality.

## 1. Introduction

Liver abscess (LA) is defined as collection of purulent material in liver parenchyma which can be due to bacterial, parasitic, fungal, or mixed infection. It is a common condition across the globe. Out of total incidence of LA, approximately two-thirds of cases in developing countries are of amoebic aetiology and three-fourths of cases in developed countries are pyogenic [1].

Amoebiasis is presently the third most common cause of death from parasitic disease [2]. The condition is endemic in tropical countries like India due to poor sanitary condition and overcrowding. Amoebic liver abscess (ALA) accounts for 3–9% of all cases of amoebiasis [3]. However, pyogenic and tubercular aetiologies should always be entertained in the differentials. The incidence of tubercular liver abscess (TLA) has increased in recent past due to increased incidence of predisposing factors like alcoholism, immunodeficiency, irrational usage of antibiotics, and emergence of drug resistant bacilli.

Surgical management was the mainstay for treating LA earlier [1]. However, recent evidences from percutaneous drainage procedure have shown a favorable outcome with less average length of stay in hospital compared to conservative mode of treatment [4]. In this context, precise diagnosis of the abscess aetiology is pivotal for appropriate management. The concept of the present study was to evaluate the changing trends in clinical profile, microbiological aetiology, and management outcomes of patients diagnosed with LA.

## 2. Material and Methods

It was a cross-sectional observational study conducted at the Department of Internal Medicine, PGIMER and Dr. Ram Manohar Lohia Hospital, New Delhi, a tertiary care centre. Study duration was from May 2011 to April 2013. Total of 200 consecutive patients diagnosed as having liver abscess on ultrasound were included in the study after taking informed written consent. Inclusion criteria were all liver abscess patients needing intervention: left lobe abscess, abscess of size >5 cms, impending rupture (<1 cm liver tissue between abscess and liver margin), and not responding to conservative management at the end of 48 hours [5–7]. Patients with age less than 18 years, organised abscess, and abscess in close proximity to large vascular structures in liver and those having pregnancy were excluded.

A detailed history, clinical examination, and laboratory profile of the patients were recorded on a predesigned Proforma. “Alcoholism” was screened as per the CAGE questionnaire [8]. Depending on the frequency of alcohol intake, patients were divided into nondrinkers, occasional drinkers (alcohol intake < 3 times/week), and regular drinkers (alcohol intake ≥ 3 times/week) [8]. Using modified Kuppuswamy's Socioeconomic Status scale, patients were divided into three socioeconomic classes: upper, middle, and lower [9]. All patients were subjected to complete hemogram, liver function test, kidney function test, and coagulation profile (PT/INR). Reference ranges of these investigations were defined by the reference ranges of hospital laboratory. Blood and urine cultures were sent. Serologies for* Entamoeba histolytica*, HIV, and hepatitis B and hepatitis C viruses were also done. All patients were subjected to Mantoux test and chest radiogram. Patients with symptoms of cough with expectoration were subjected to sputum for acid fast bacilli (AFB) using ZN staining to rule out pulmonary Koch's.

**Figure 1:**
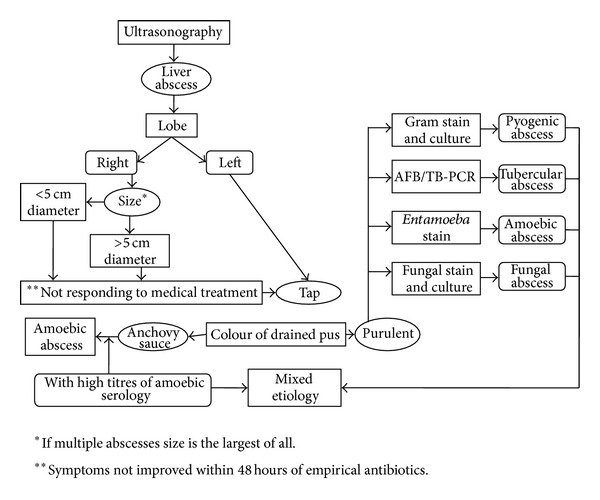
Flowchart depicting protocol followed for percutaneous ultrasound guided drainage of liver abscess and its appropriate processing [7, 8].

After taking informed consent, all patients were subjected to ultrasound guided aspiration of liver abscess either by percutaneous needle or pigtail catheter. Interventions were done after correction of INR below 1.4 to those who presented with coagulopathy. We preferred pigtail catheter in single, large (>10 cm), deep seated, and partially liquefied abscess. In multiple, small (5–10 cm), superficial, and fully liquefied abscesses, we tend to use percutaneous catheter. Aspirate was collected in sterile containers and sent immediately to Microbiology Department for microscopic examination of wet mount for trophozoites of* Entamoeba histolytica*, Gram's staining, and ZN staining for AFB. Samples were plated in aerobic, anaerobic, and fungal culture media. Till pus culture report was received, patients were empirically started on intravenous ceftriaxone and metronidazole. Discharge criteria were considered as normalisation of hemodynamic status with defervescence of the presenting complaints. Protocol of management followed by us has been shown in Figure 1 [7, 8, 10, 11].

All data were collected in MS-excel sheet and analysed using statistical software package SPSS version 19. Mean, median, and standard deviation were calculated for continuous variables. Chi-square test and multivariate regression analysis were used for test of association.

## 3. Results

A total of 200 patients with liver abscess were studied and analysed. The mean age of the patients was 41.13 years (range: 19 to 78 years). Male to female ratio was 13.3 : 1. About two-thirds of the patients (67.5%; *n* = 135) were from lower socioeconomic class with regards to education, occupation, and per capita income and the rest were from the middle class families.

Pain abdomen was the most common symptom (99%; *n* = 198). Tender hepatomegaly was the most common per-abdominal examination finding (Table 1). Pleural effusion was evident in 30% (*n* = 60) of the patients, predominantly on the right side (23%; *n* = 46); however, left-sided and bilateral effusions were also encountered in 3% and 4% of patients, respectively. HIV was reactive in only 2% of patients and viral markers (HBsAg and anti-HCV) were nonreactive in all patients. The laboratory profile of the patients is mentioned in Table 2.

Involvement of right lobe in these cases was most predominant (71%). The same is true for solitary presentation (65%). The findings on ultrasonography of the abdomen are depicted in Table 3. About 6% of the patients also had evidence of typhlitis at presentation.

For management (Table 4), 79% (*n* = 158) of the patients had percutaneous needle aspiration of their abscess content and 17% (*n* = 34) underwent pigtail drainage. Surgical intervention was done in 8 (4%) patients for managing rupture. Out of them, 5 died; thus, overall mortality was 2.5%.

**Figure 2:**
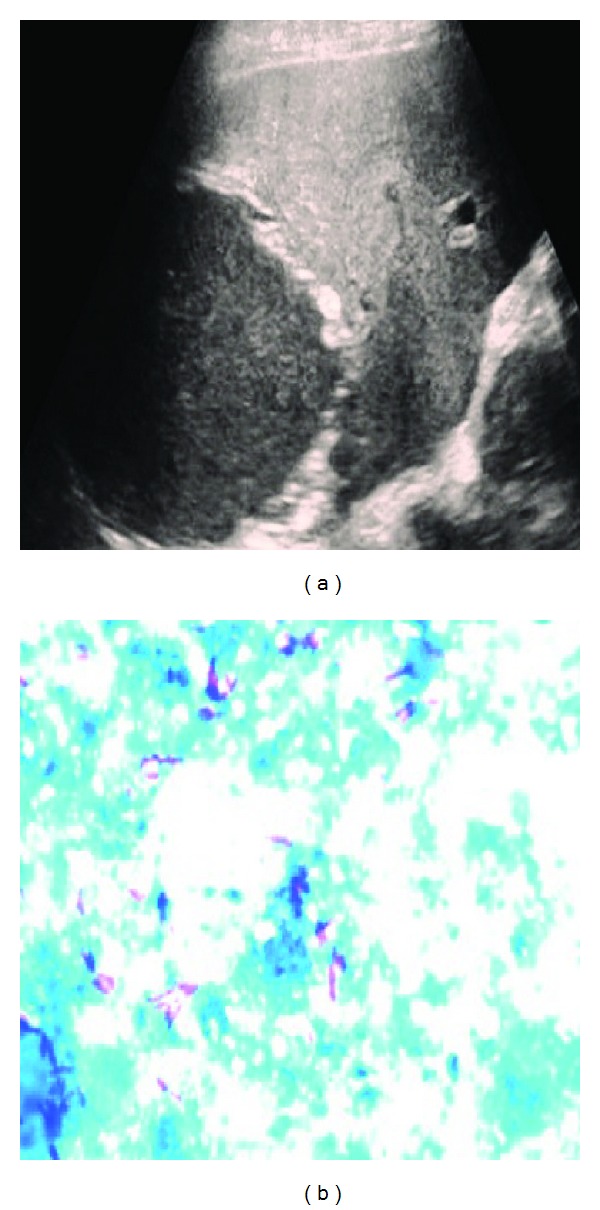
Tubercular liver abscess. (a) Ultrasonography image of a large hypoechoic lesion involving the right lobe of liver and (b) aspirated pus on ZN staining depicts acid fast bacilli.

Etiological analysis of LA revealed that 69% were of amoebic origin (*n* = 138), 18% of pyogenic (*n* = 36), 4% of mixed amoebic and pyogenic process (*n* = 8), 7.5% of tubercular (*n* = 15), and 1.5% of fungal infections (*n* = 3) (Table 5).  Figure 2 depicts a solitary LA which was drained and pus came out to be positive for AFB. Amoebic serology was positive for IgM antibodies with significant titres in 72.5% (*n* = 145) patients. Pus culture gave positive results in 22% (*n* = 44) of the patients, which grew predominantly Gram negative flora. Blood and urine cultures were however positive in only 1.5% and 3% of the patients, respectively. According to culture reports from that of blood and pus, change in antibiotics was done in 25% (*n* = 50) of the patients.

Solitary abscess was more of amoebic and tubercular in etiology whereas multiple abscesses were associated with pyogenic origin (*P* = 0.001). Amoebic and pyogenic liver abscesses were more frequent in the right lobe and tubercular in left lobe (*P* = 0.001). ALA patients were found to be more frequently alcoholic (*P* = 0.013) and had greater weight loss (*P* = 0.008). TLA was more commonly associated with ascites (*P* = 0.002).

Using multivariate regression analysis, volume of abscess was found to be directly proportional to the levels of serum alkaline phosphatase (*P* = 0.041) and inversely to haemoglobin (*P* = 0.005) levels of the patient. Duration of hospitalisation as a morbidity indicator was proportional to duration of fever (*P* = 0.02), values of ESR (*P* = 0.021), and INR (*P* = 0.043). It was inversely related to serum albumin (*P* = 0.033). Mortality rates were found higher in female patients (*P* = 0.001), patients having longer duration of fever (*P* = 0.001), icterus (*P* = 0.001), ascites (*P* = 0.006), and pleural effusion (*P* = 0.028) (Table 6). The detailed statistical analysis has been provided as supplementary (see Tables A to F in Supplementary Material available online at http://dx.doi.org/10.1155/2014/142382) in a separate file.

## 4. Discussion

Liver abscess (LA) is common in the tropical region like the Indian subcontinent. The common etiological agents for LA are* E. histolytica* (amoebic), bacterial (pyogenic), Mycobacterium tuberculosis, and various fungi. Out of them, ALA is largely a disease of developing countries like India. They tend to affect younger population especially males. Common presenting complains are abdominal pain, fever, and weight loss. It is also an important cause of fever of unknown origin. Coexisting diarrhoea occurs in 30% of patients and it is extremely rare to find amoebic trophozoites in the stool examination [12].

In our series also ALA accounted for about three-fourths of cases. Most of them were typically right lobe solitary abscess. This pattern of involvement has also been reported in previous series on ALA like by Sharma et al. [7] and Mukhopadhyay et al. [13]. Majority of patients were young alcoholic male (with mean age of 41 years) of lower socioeconomic class which is also in accordance with the previous studies [13]. The age predisposition and gender differences may be as a result of high alcohol intake by young male which predisposes to ALA. Alcohol suppresses function of Kupffer cells (specialized macrophage) in liver which has important role in clearing amoeba. Moreover, invasive amoebiasis appears to be dependent on the availability of free iron. A high content of iron in the diet, often obtained from the country liquor in habitual drinkers predisposes to invasive amoebiasis, as does a diet rich in carbohydrate [14]. Elderly individuals with underlying diseases and patients with compromised immunity due to malnutrition or corticosteroid therapy are also prone to invasion by amoeba. Moreover, Reddy and Thangavelu proposed that the female menstrual cycle prevents hepatic congestion and thus makes the organ less susceptible to abscess formation [15].

As amoebic liver abscesses are uncommon due to good hygiene in region with temperate climate, pyogenic liver abscesses (PLA) are etiologically more common in west. In a large series by Ochsner et al. [1], the disease was described as primarily affecting young male patients in the setting of intra-abdominal infections and reported high mortality with nonoperative treatment and multiple abscesses. Since then, with effective treatment of predisposing intra-abdominal conditions, there has been a decreasing trend in mortality in the young and a subsequent increased incidence in the elderly age group. Generally, PLA are associated with predisposing benign or malignant biliary tract or colonic disease: acute cholecystitis, choledocholithiasis, biliary-enteric bypass procedures, chronic pancreatitis, diverticulitis, colonic perforation, appendiceal abscess, perforated appendicitis, malignant obstruction of the common bile duct, cholangiocarcinoma, pancreatic carcinoma, and carcinoma of the colon [12]. However, recent trend is towards the increase in the frequency of patients with cryptogenic PLA in which no specific lesion predisposing to PLA could be identified even after detailed search [12, 16]. In our series, PLA accounted for about quarter of cases. Most of them were multiple and right lobe abscesses. Average age in this group was not different from overall average (43.27 years compared to 41 years overall). In the study by Pang et al. [17] and Heneghan et al. [18] among PLA patients, average age of presentation was 65 and 60.3 years, respectively. Etiologically, Gram negative organisms commonly inhabiting the gut and biliary microflora were frequently encountered by us,* E. coli* being the most common pathogen. It was in line with previous experiences [17].

Third pattern we noted was that of tubercular liver abscess. Tubercular involvement of liver is uncommon as the low oxygen level in liver is unfavorable for TB bacilli to survive. Generally, it has a primary focus in lung or gastrointestinal tract (GIT), from where it spreads to the liver as a part of haematogenous dissemination via hepatic artery and portal vein, respectively. Based on morphological pattern of involvement, Reed et al. [19] classified hepatic TB into three patterns: primary miliary TB of liver (diffuse liver involvement without evidence of TB anywhere else); diffuse liver involvement with pulmonary TB; and focal lesion presenting as abscess. Latter one is the most uncommon pattern of hepatic tuberculosis. Isolated TLA is the rarest. Essop et al. [20] found TLA in only 0.34% of patients with hepatic TB. In our patients with TLA, most of cases were solitary and in left lobe. They were commonly associated with ascites. Interestingly, all were AFB positive. The fourth pattern in our series was fungal abscess. They are the rare cause of LA. We reported 3 fungal abscesses, all grew* Candida*. Two of these patients were HIV positive and one was diabetic.

Our demographic data had some interesting trends. Mean age in our series was 41 years, which was in accordance with Indian studies like by Sharma et al. [7] and Mukhopadhyay et al. [13] who reported it to be 40.5 and 43.64 years, respectively. It is because ALA is the predominant aetiology in the Indian scenario, typically involving young alcoholics. In contrast, studies from west where PLA are more common, average age is above 60 years [17, 18, 21]. However, Giorgio et al. [22] in group of PLA patients reported average age to be 45.3 years. As far as sex predisposition was concerned, even after recruiting 200 consecutive patients, only 13 patients were female. Indian data show predominant male involvement; Sharma et al. [7] and Mukhopadhyay et al. [13] reported male to female ratio to be 7 : 1 and 11 : 1, respectively. However, Pang et al. [17] and Heneghan et al. [18] reported it to be 2 : 1 and 1.22 : 1, respectively. Thus, as age increases, risk of pyogenic abscess and female patients getting LA increases. Two-thirds of our patients were from lower socioeconomic class. All of them were alcoholic and thus predisposed to LA. In the study by Mukhopadhyay et al. [13], 61.11% of patients were alcoholic.

Most common symptoms of LA are pain abdomen and fever which were present in 99% and 94% of our patients, respectively. Various studies quote it in range of 62–94% and 67–87%, respectively [7, 13]. Diarrhoea in LA could be due to associated intestinal amoebiasis and could be part of colonic condition predisposing to LA. It is not a common presentation; we reported it in 23% of patients. Previous studies report it variably from 4% to 33% [7, 13, 23–25]. Another uncommon complain in LA is cough. It is generally due to associated pleural effusion and compression collapse of the underlying lung parenchyma. Other causes are associated parenchymal lesions as in TLA and complications like rupture of abscess in pleural cavity. Cough as a symptom in our patients was present in 16% of patient. Previous series report it in 3.5–24% of cases [7, 26]. Pleural effusion was present in 30% of our patients; all patients with cough belong to this group. Chest radiography helped a little with the diagnosis of LA, except for raised right hemidiaphragm giving some indirect clue of hepatomegaly. Most importantly, they showed associated pleural effusion which was predominantly right-sided in most of our cases. The effusion was generally attributed to reactive pathology as they spontaneously disappeared after treating the abscess.

Two uncommon signs of LA are jaundice and ascites. Jaundice was seen in 26% of our patients. In earlier studies from India, it was reported in 45–50% of patients [27]. But after advent of good antimicrobial therapy, it has become less common. Sharma et al. reported it in only 12.7% of patients [7]. Yoo et al. [28] in their study compared data of patients between 1970s and 1980s and reported a fall in incidence of jaundice from 25% to 7% during this period. Pathogenic processes proposed which can lead to jaundice are sepsis, alcoholic liver disease, hepatocellular dysfunction, associated hepatitis in the adjoining areas, intrahepatic biliary obstruction by the expanding abscess, and biliovascular fistula resulting from hepatic necrosis leading to damage of bile ducts and hepatic veins [29, 30]. However, no biliovascular fistula was detected by ultrasound doppler in any of our case. The other sign infrequently associated with LA was ascites. It was present in 18 patients out of which 5 had TLA and 8 had associated decompensated chronic liver disease (CLD). Apart from it, cases have been reported where LA cause ascites by compressing the inferior vena cava [31]. Nigam et al. reported ascites in 10.5% of patients [32]. So presence of ascites should raise suspicion of TLA or associated CLD.

Abdominal ultrasound is still the diagnostic modality of choice for hepatic pathologies including LA. Its sensitivity to detect the LA ranges from 92 to 97% [24, 33]. We used transducer of 3 MHz frequency for localising the abscess and hepatic vasculature. One can localise the specific segment involved with high accuracy by determining the location of abscess with respect to hepatic vasculature [34]. Saggital plane scan precisely define the segment involved and accurately localise the abscess. Operator expertise is also necessary for achieving the high accuracy [34]. In our study, 6th and 7th segments in right lobe were most commonly involved. The predilection of LA in right lobe is because of streaming effect in portal circulation [13, 35]. It receives most of blood draining from right colon, the primary site of intestinal amoebiasis. Colonic conditions predisposing to PLA are also very common in this region. Also, the blood flow volume is more and biliary canaliculi are denser in right lobe thus leading to more congestion [35].

Consistent with the latest management strategy of minimally invasive drainage techniques, percutaneous needle aspiration was used in most of patients (79%) [36]. Appropriate antimicrobials were added according to the etiological outcome. However, 4% of the patients had to undergo surgical intervention as they got complicated by rupture. Other possible indications for surgical intervention could be inaccessible anatomical location, failure to response to treatment after conservative therapy, and other associated complications like peritonitis, biliary-enteric fistulisation, and so forth. All mortality was reported in these patients with surgical intervention. Overall, it was 2.5%, which was similar to previous series where it has been recorded between 2 and 15% [7, 26]. Interestingly, average age in this group was 64.8 years (range: 45 to 78 years), which was quite less compared to overall average of 41 years. Other details of these patients were as follows: 3 of them were female; 2 ALA, 2 PLA, and 1TLA all of them had icterus and hepatomegaly; 4 patients had pallor; 3 had ascites; and 4 had pleural effusion. Laboratory results had greater derangements (mean values): ESR 31 mm in 1st hour, haemoglobin 9 gm/dL, TLC 37300/mL, urea 161 mg/dL, bilirubin 4.9 mg/dL, albumin 2.4 g/dL, SGOT 232 IU/L, SGPT 203 IU/L, ALP 1562 IU/L, and INR of 1.77. On ultrasound, 4 out of 5 were solitary abscess in the right lobe with mean abscess volume of 382 cc. Mean duration of hospital stay, that is, onset of hospitalisation to mortality duration, was 7.2 days, ranging from 3 to 11 days. Mortality indicators proved to be sex of the patient with female patients with lower survival as also with longer duration of fever before presenting to medical facility and presence of icterus, ascites, and pleural effusion.

## 5. Conclusions

Young alcoholic male from lower socioeconomic group with amoebic liver abscess presenting as solitary right lobe abscess was the most common pattern in our series. Liver abscess was uncommon in female patients. Apart from amoebic and pyogenic, tubercular liver abscesses were not so uncommon etiologically. Though average age of patients was in forties, increased incidence of mortality was noted in patients in the seventh decade. Cough as a symptom points to associated significant pleural effusion. Presence of ascites should raise suspicion of TLA or associated CLD. Mortality was high in patients undergoing surgical intervention for rupture. Overall mortality was low probably due to use of minimally invasive drainage techniques and aetiology specific antimicrobials in all patients.

## Supplementary Material

Statistical analysis: Appropriate statistical analysis was done between clinically independent variables and outcome parameters to test for significance (*p*<0.05). Chi square test was applied in Table A which shows that solitary right sided LA in alcoholic patients were significantly amebic in etiology; whereas left sided abscess with ascites and associated weight loss pointed more towards tubercular origin. Chi square test analysis in Table B shows that abscess involving both lobes of liver have increased incidence of pleural effusion. In Table C, non-invasive mode of drainage of LA was analyzed, which showed that large abscess causing hepatomegaly were better drained by pig tail catheterization; however percutaneous needle aspiration had a better outcome in right-sided LA. In Table D, prognosis was evaluated using chi square test which revealed incidence of death was high in female patients, patients who had fever, icterus, ascites and pleural effusion. Continuous variables like abscess volume and duration of hospitalization was evaluated with correlation and regression statistics. In Table E, Spearman's rank correlation and multivariate regression analysis was used to assess factors contributing to the volume of abscess measured on ultrasound. TLC, SGOT, SGPT, ALP levels and INR value were found to be directly proportional to abscess volume. In Table F, duration of hospitalization in all patients of LA was similarly analyzed. ESR, blood urea, bilirubin SGOT, ALP levels and INR value were directly proportional to the total number of days of hospitalization.

## Figures and Tables

**Table 1:** Clinical profile of patients: pain abdomen and fever were the two most common symptoms while hepatomegaly was the most frequent clinical finding.

	Parameters	Percentage (=*n*)
Symptoms	Pain abdomen	99% (198)
	Fever	94% (188)
	Anorexia	93% (186)
	Nausea/vomiting	54% (108)
	Diarrhea	23% (46)
	Cough	16% (32)
	Weight loss	40% (80)

Risk Factors	Alcoholic Diabetic	72% (144) 9% (18)

Signs	Pallor	39% (78)
	Jaundice	26% (52)
	Hepatomegaly	89% (178)
	Splenomegaly	10% (20)
	Ascites	9% (18)
	Pleural effusion	30% (60)

**Table 2:** Laboratory investigations: all parameters, particularly those related to liver were deranged in good percentage of patients. Sepsis indicators like raised TLC and low albumin were present in more than three-fourths of patients.

Parameters	Mean ± SD	Out of range cut off	Out of range percentage
ESR	44 ± 28 mm 1st hr	>20 mm in 1st hour	72%
Hb %	11.2 ± 1.9 gm/dL	<11 gm/dL	40.5%
TLC	19,100 ± 9104/*μ*L	>11000/*μ*L	82%
INR	1.37 ± 0.25	>1.2	75%
Bilirubin	1.55 ± 2.18 mg/dL	>1.2 mg/dL	27%
SGOT	83 ± 137 IU/L	>50 IU/L	47%
SGPT	62 ± 67 IU/L	>50 IU/L	42%
Alkaline phosphatase	622 ± 446 IU/L	300 IU/L	79%
Albumin	3.0 ± 0.56 g/dL	<3.5 g/dL	82%
Urea	40 ± 36.8 mg/dL	>45 mg/dL	27%
Calcium	8.21 ± 0.8 mg/dL	<8 mg/dL	35%

**Table 3:** Ultrasonography abdomen findings for liver abscess: right lobe solitary abscess was the most common pattern. Segments 7 and 6 were the most common sites of abscess. Associated typhlitis, an uncommon finding with ALA, was present in only 6% patients.

	Parameters	Percentage (=*n*)
Lobe	Right	71% (142)
	Left	17.5% (35)
	Bilateral	11.5% (23)

Number	Solitary	65% (130)
	Few (≤3)	11% (22)
	Multiple (>3)	23.5% (47)

Typhlitis		6% (12)

Mean abscess vol. ± SD		270 ± 205 cc

Segment involved	VII	35% (70)
	VI	25% (50)
	VIII	10% (20)
	V	10% (20)
	IV	10% (20)
	Rest	10% (20)

**Table 4:** Management outcome: majority of patients were managed by needle aspiration. Surgical intervention was done in 8 patients, all for rupture, out of which 5 died.

	Parameters	Percentage (=*n*)
Abscess drainage	Percutaneous needle aspiration	79% (158)
	Pigtail drainage	17% (34)
	Open surgical	4% (8)

Change of antimicrobials required		25% (50)

Mean duration of	Hospitalisation	8 ± 5.36 days
	Treatment	33 ± 42 days

Surgical intervention		4% (8)

Mortality		2.5% (5)

**Table 5:** Etiological analysis: amoebic serology was positive in 73% patients and, in accordance, aspirate was anchovy sauce in 71% of them. All cases of diagnosed tubercular abscess were AFB positive. In pyogenic liver abscess, Gram negative gut flora predominated etiologically.

	Parameter	Percentage (*n*)
Appearance	Anchovy sauce	71% (142)
	Purulent	29% (58)

Amoebic serology positive		73% (146)

AFB positivity on pus		7.5% (15)

Fungal culture on pus	*Candida *	1.5% (3)

Positive cultures on pus		22% (44)

Etiological agents in positive pus culture	*E.coli *	8.5% (17)
	*Klebsiella *	5.5% (11)
	*Pseudomonas *	2% (4)
	*Acinetobacter *	2% (4)
	*Staphylococcus *	2% (4)
	*Enterococcus *	1.5% (3)
	*Citrobacter *	0.5% (1)

Blood culture positive		1.5% (3)

**Table 6:** Statistical analysis: results of statistical analysis of abscess size and duration of hospitalisation with various clinical and laboratory variables using multivariate analysis. Association of these variables with mortality was studied using Chi-square test, which has also been given (only data with significant associations has been given).

	Parameter	Correlation coefficient	*P* value
Abscess volume	Anemia	−0.33	0.005
	Alkaline phosphatase	0.37	0.041

Duration of hospitalisation (morbidity indicators)	Duration of fever		0.02
	ESR	0.16	0.021
	INR	0.20	0.043
	Albumin	−0.28	0.033

Mortality	Females		0.001
	Duration of fever		0.001
	Icterus		0.001
	Ascites		0.006
	Pleural effusion		0.028
