# Supplementary material for: Clinical, Laboratory, and Management Profile in Patients of Liver Abscess from Northern India
Source: J Trop Med. 2014 Jun 4;2014:142382. doi: 10.1155/2014/142382 (PMC4066852; doi:10.1155/2014/142382)
Supplement: Supplementary file 1 — Statistical analysis: Appropriate statistical analysis was done between clinically independent variables and outcome parameters to test for significance (p<0.05). Chi square test was applied in Table A which shows that solitary right sided LA in alcoholic patients were significantly amebic in etiology; whereas left sided abscess with ascites and associated weight loss pointed more towards tubercular origin. Chi square test analysis in Table B shows that abscess involving both lobes of liver have increased incidence of pleural effusion. In Table C, non-invasive mode of drainage of LA was analyzed, which showed that large abscess causing hepatomegaly were better drained by pig tail catheterization; however percutaneous needle aspiration had a better outcome in right-sided LA. In Table D, prognosis was evaluated using chi square test which revealed incidence of death was high in female patients, patients who had fever, icterus, ascites and pleural effusion. Continuous variables like abscess volume and duration of hospitalization was evaluated with correlation and regression statistics. In Table E, Spearman's rank correlation and multivariate regression analysis was used to assess factors contributing to the volume of abscess measured on ultrasound. TLC, SGOT, SGPT, ALP levels and INR value were found to be directly proportional to abscess volume. In Table F, duration of hospitalization in all patients of LA was similarly analyzed. ESR, blood urea, bilirubin SGOT, ALP levels and INR value were directly proportional to the total number of days of hospitalization. [file 142382.f1.pdf]

Variables		Abscess aetiology				P value
		Amoebic	Pyogenic	Tubercular	Others	
No. of abscess	Solitary	104	10	12	5	0.001
	Few (2-3)	16	4	1	1	
	Multiple	18	22	2	5	
Alcoholic	Yes	107	20	7	9	0.013
	No	31	16	8	2	
Weight loss	Yes	49	13	12	7	0.008
	No	89	23	3	4	
Ascites	Yes	7	5	5	1	0.002
	No	131	31	10	10	
Liver lobe involved	Right	106	18	9	9	0.001
	Left	26	4	5	0	
	Both	6	14	1	2	

Table A: chi square test analysis between liver abscess etiology and different variables.

Variables		Pleural effusion				P value
		No	Right	Left	Bilateral	
Liver lobe involved	Right	101	34	4	3	0.009
	Left	25	8	1	1	
	Both	11	8	0	4	
No. of abscess	Solitary	100	25	4	3	0.101
	Few (2-3)	9	12	0	1	
	Multiple	28	13	1	4	

Table B: chi square test analysis between incidence of pleural effusion and USG features of liver abscess

Variables		Mode of Drainage		P value
		Percutaneous	Pigtail Cath	
Hepatomegaly	Yes	139	34	0.05
	No	22	0	
Liver lobe involved	Right	127	29	0.022
	Left	15	5	
	Both	19	0	

Table C: chi square test analysis of mode of drainage with hepatomegaly and the lobe of liver affected.

Variables		Prognosis		P value
		Discharged	Death	
**Sex**	**Male**	184	3	0.001
	**Female**	11	2	
**Fever**	**Yes**	185	3	0.001
	**No**	10	2	
**Alcoholic**	**Yes**	141	2	0.114
	**No**	54	3	
**Pallor**	**Yes**	74	4	0.057
	**No**	121	1	
**Icterus**	**Yes**	46	5	0.001
	**No**	149	0	
**Ascites**	**Yes**	15	3	0.006
	**No**	180	2	
**Pleural effusion**	**No**	139	1	0.028
	**Right**	43	4	
	**Left**	5	0	
	**Bilateral**	8	0	
**Liver lobe involved**	**Right**	138	4	0.71
	**Left**	34	1	
	**Both**	23	0	
**Mode of abscess drainage**	**None**	5	0	0.37
	**Percutaneous**	158	3	
	**Pig tail Cath.**	32	2	
**Etiology**	**Amoebic**	136	2	0.50
	**Pyogenic**	34	2	
	**Tubercular**	14	1	
	**Others**	11	0	

Table D: chi square test analysis between the prognosis and various clinical parameters.

Abscess volume	Correlation		Regression		
**Variables**	**r (corr. Coeff.)**	**p value**	**B**	**Std. Error**	**Sign. (p value)**
**Age**	- 0.07	0.302	-1.255	1.102	0.256
**ESR**	- 0.43	0.55	-0.426	0.481	0.377
**Haemoglobin**	- 0.33	0.001	-22.410	7.814	0.005
**TLC**	0.15	0.032	0.001	0.002	0.646
**MCV**	0.04	0.56	1.602	1.205	0.186
**Urea**	0.14	0.054	-0.562	0.492	0.254
**Bilirubin**	0.09	0.185	-3.518	7.526	0.641
**Albumin**	- 0.29	0.001	-50.594	29.716	0.090
**SGOT**	0.23	0.001	0.072	0.146	0.625
**SGPT**	0.19	0.007	0.504	0.316	0.112
**ALP**	0.37	0.001	0.075	0.036	0.041
**Calcium**	- 0.21	0.003	-10.591	19.964	0.596
**INR**	0.27	0.001	89.563	65.371	0.172

Table E: Composite spearman's rank correlation and multivartiate regression analysis between abscess volume and various clinic-pathological parameters.

Duration of Hospitalisation	Correlation		Regression		
**Variables**	**r (corr. Coeff.)**	**p value**	**B**	**Std. error**	**Sign. (p value)**
**Age**	0.02	0.831	- 0.007	0.030	0.808
**Duration of fever**	0.09	0.232	0.143	0.061	0.020
**Duration of pain abdo**	- 0.08	0.233	-0.123	0.064	0.055
**ESR**	0.16	0.017	0.030	0.013	0.021
**Hemoglobin**	- 0.14	0.040	- 0.125	0.216	0.563
**TLC**	0.15	0.035	4.94	0.001	0.919
**MCV**	- 0.13	0.075	- 0.058	0.033	0.078
**Urea**	0.19	0.008	- 0.007	0.013	0.621
**Bilirubin**	0.14	0.045	0.250	0.204	0.221
**Albumin**	- 0.28	0.001	- 1.739	0.811	0.033
**SGOT**	0.21	0.003	0.004	0.004	0.310
**SGPT**	0.10	0.174	- 0.015	0.009	0.078
**ALP**	0.17	0.018	0.001	0.001	0.422
**Calcium**	- 0.15	0.031	- 0.235	0.541	0.664
**INR**	0.20	0.005	3.619	1.779	0.043
**Abscess volume**	0.11	0.123	- 0.002	0.002	0.320

Table F: Composite spearman's rank correlation and multivartiate regression analysis between duration of hospitalisation and various clinic-pathological parameters.
